# IL-17RA Is Required for CCL2 Expression, Macrophage Recruitment, and Emphysema in Response to Cigarette Smoke

**DOI:** 10.1371/journal.pone.0020333

**Published:** 2011-05-27

**Authors:** Kong Chen, Derek A. Pociask, Jeremy P. McAleer, Yvonne R. Chan, John F. Alcorn, James L. Kreindler, Matthew R. Keyser, Steven D. Shapiro, A. McGarry Houghton, Jay K. Kolls, Mingquan Zheng

**Affiliations:** 1 Department of Genetics, Louisiana State University Health Science Center, New Orleans, Louisiana, United States of America; 2 Department of Medicine, University of Pittsburgh School of Medicine, Pittsburgh, Pennsylvania, United States of America; 3 Children's Hospital of Philadelphia, Philadelphia, Pennsylvania, United States of America; 4 DNASTAR, Madison, Wisconsin, United States of America; New York University, United States of America

## Abstract

Chronic Obstructive Pulmonary Disease (COPD) is characterized by airspace enlargement and peribronchial lymphoid follicles; however, the immunological mechanisms leading to these pathologic changes remain undefined. Here we show that cigarette smoke is a selective adjuvant that augments *in vitro* and *in vivo* Th17, but not Th1, cell differentiation via the aryl hydrocarbon receptor. Smoke exposed IL-17RA^−/−^ mice failed to induce CCL2 and MMP12 compared to WT mice. Remarkably, in contrast to WT mice, IL-17RA^−/−^ mice failed to develop emphysema after 6 months of cigarette smoke exposure. Taken together, these data demonstrate that cigarette smoke is a potent Th17 adjuvant and that IL-17RA signaling is required for chemokine expression necessary for MMP12 induction and tissue emphysema.

## Introduction

Chronic obstructive pulmonary disease (COPD) accounts for 5–10% of all deaths globally and it is estimated that 80 million people currently suffer with COPD of varying degrees [1]. COPD is attributed to persistent exposure to noxious gases and particulates, and is most often associated with cigarette smoke. This exposure induces a chronic inflammatory state in which there is mucus hypersecretion and the formation of airway associated lymphoid follicles [1]–[3]. This leads to inflammatory obstruction of the upper and lower airways and destruction of parenchymal structure (emphysema), leading to chronic, progressive decrease in airflow [2], [3].

T lymphocytes are a prominent cell type in the COPD inflammatory milieu. CD4^+^ and CD8^+^ T cells are found in both airways and parenchyma during COPD and increases in these populations is associated with a greater severity of disease and worsening prognosis [3], [4]. COPD-associated T cells produce Th1 [5]–[7] and Th2 cytokines [8]. Recently, IL-17 producing T cells known as Th17 cells have been identified in lungs from COPD patients [4], [9], suggesting an important role for this cytokine.

T helper 17 (Th17) cells are a critical component of the adaptive immune response, but have also been implicated in chronic inflammatory diseases and autoimmune diseases [10]. Naive T cells differentiate into Th17 cells through co-exposure to IL-6 and TGF-β, both of which are present during COPD. It has also been shown that the aryl hydrocarbon receptor (AhR), which is activated by cigarette smoke, may have a role in promoting the Th17 response and expression of IL-22 [11]. Moreover, IL-17A was recently detected in sputum from patients with COPD and this correlated with airflow obstruction [12], [13]. Interestingly, tobacco smoke is associated with autoimmune diseases, particularly systemic lupus erythematosus [14], rheumatoid arthritis [15] and inflammatory bowel diseases [16]. While these studies demonstrate the presence of IL-17 in COPD, whether Th17 cells or their cytokine products contribute to pathology remains unknown.

In the study, we show that cigarette smoke is a selective adjuvant that augments mucosal Th17 but not Th1 cell differentiation *in vitro* and *in vivo* in part by an aryl hydrocarbon receptor-dependent mechanism. RNA sequencing analysis of human bronchial epithelium shows that IL-17 and IL-22 can induce MMP3 and MMP12, the latter gene implicated in cigarette smoke-induced emphysema [17]. Mice lacking IL-17RA showed substantially reduced macrophage recruitment, CCL2 and CXCL10 in bronchoalveolar lavage after 6 months of smoke exposure. This reduced macrophage recruitment was associated with reduced expression of MMP12 transcripts in the lung. Remarkably, in contrast to WT mice, IL-17RA^−/−^ failed to develop emphysema after 6 months of cigarette smoke exposure. Taken together these data demonstrate that cigarette smoke is a potent Th17 adjuvant and that IL-17RA signaling is required for the elaboration of CCL2, macrophage recruitment and tissue emphysema. These data suggest a novel immune pathway that may be a target of intervention for COPD.

## Results

### Cigarette Smoke is a Th17 Adjuvant

**Figure 1:**
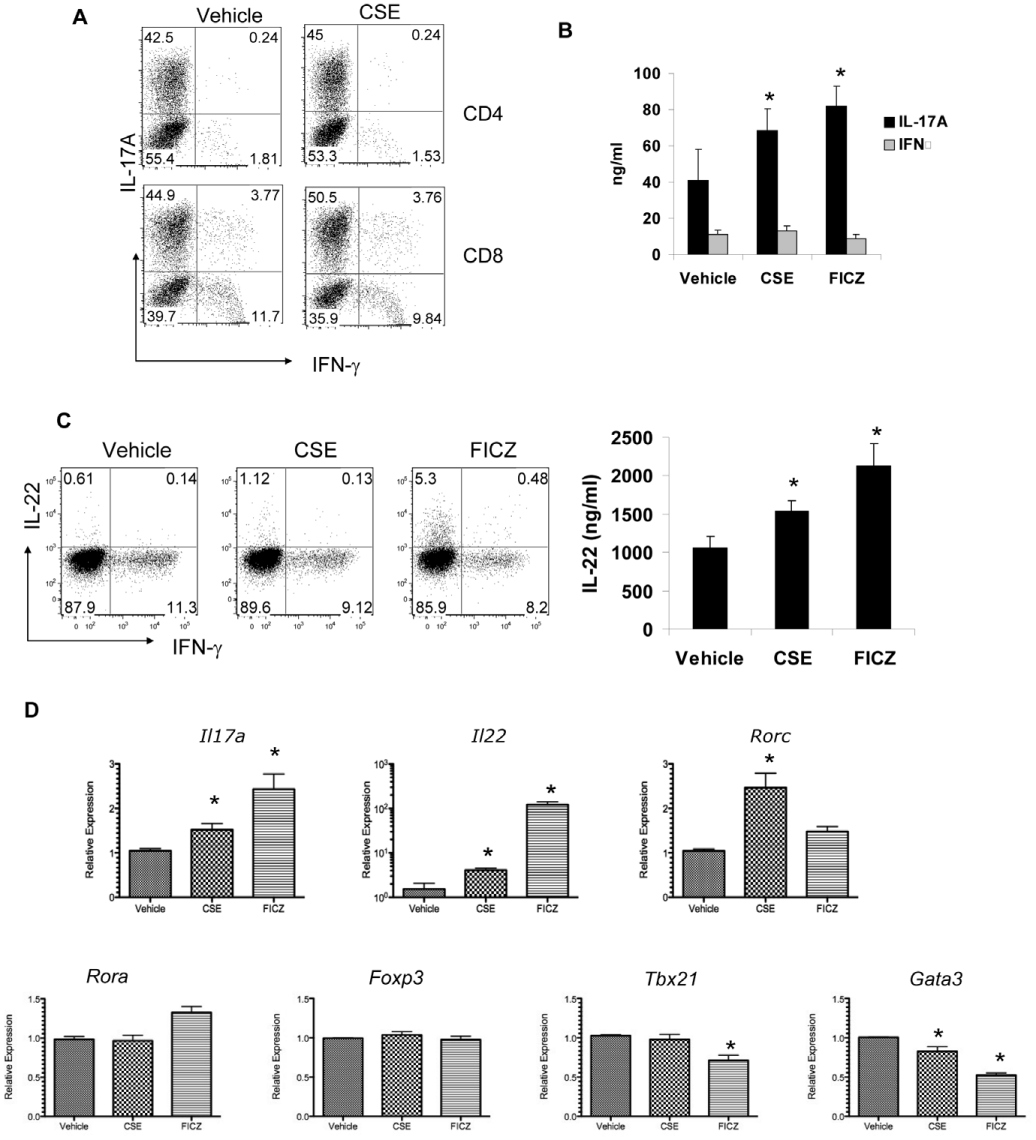
Cigarette smoke extract enhances *in vitro* Th17 polarization. Naïve CD4+ or CD8+T cells from C57BL/6 spleen were activated by plate bound anti-CD3 and anti-CD28 in the presence of TGF-β, IL-6, anti-IFN-γ and anti-IL4. Where indicated, 0.5% CSE or 200nM FICZ were added into the culture. On day 3, cells were harvested and intracellular cytokines were analyzed by FACS (A, C). Tissue culture supernatants were harvested from *in vitro* polarized Th17 cells for secreted IL-17 by Luminex (C, n = 5 per group, * denotes p<0.05 compared to vehicle). RNA was extracted from the same cells for gene expressions analysis by real-time RT-PCR (D, n = 5 per group, * denotes p<0.05 compared to vehicle-HBSS).

Cigarette smoke is a complex mixture of gaseous and particulate materials that contains Ahr ligands as well as LPS [18], [19] and has been shown to induce IL-23 in macrophages [20]. To examine if cigarette smoke affects Th17 differentiation, we purified naïve CD4^+^ and CD8^+^ T-cells from the spleens of C57BL/6 mice and performed T-cell differentiation under Th17 polarizing conditions in the presence or absence of cigarette smoke extract (CSE). The addition of 0.5% CSE significantly increased the percentage of CD4 and CD8 T-cells that stained positive for IL-17 (Figure 1A). Moreover, Luminex analysis of cell supernatants also showed that these cells produce more secreted IL-17 in culture (Figure 1B). CSE also increased IL-22 expression during Th17 polarization in RPMI 1640 media which lacks Ahr ligands [21] (Figure 1C). As a positive control the Ahr ligand FICZ also stimulated IL-22 production. Addition of CSE substantially increased *Rorc*, a critical transcription factor for Th17 differentiation, in addition to *Il17a* and *Il22* (Figure 1D). Again FICZ also increased transcripts for *Il17a* and *Il22* in this culture system (Figure 1D). Neither CSE nor FICZ affected the expression of *Foxp3.* However FICZ suppressed *Tbx21* expression and both FICZ and CSE suppressed expression of *Gata3* (Figure 1D).

**Figure 2:**
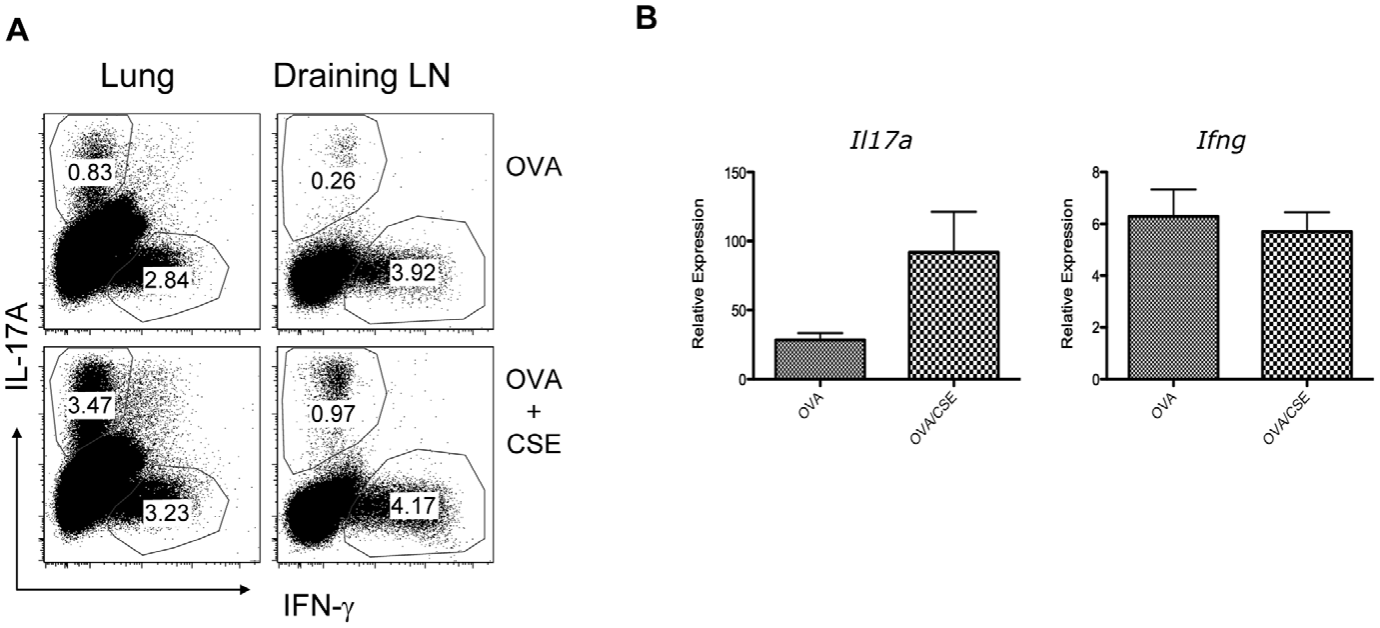
Cigarette smoke extract enhances Th17 differentiation *in vivo*. (A) OTII mice were given 50 µg OVA in 50 µL HBSS or CSE intratracheally on day 0, day 7 and sacrificed on day 14. Single cell suspensions harvested from lung digest and mediastinal lymph nodes were re-stimulated with PMA and ionomycin in the presence of Golgi-plug for 5h, intracellular cytokines were analyzed by FACS (A). Gene expression in lung tissue was analyzed by real-time RT-PCR (B, n = 5 per group, * denotes p<0.05 compared to OVA alone).

We next examined if CSE could serve as a Th17 adjuvant *in vivo*. For these studies we administered ovalbumin (OVA) intratracheally to OVA specific T-cell receptor transgenic (OT-II) mice with or without CSE. Sevens days later, Th priming was assessed in lung and mediastinal lymph nodes by FACS. OVA alone induced modest numbers of Th17 and Th1 cells in both the lung and mediastinal lymph node tissues (Figure 2A). However, the addition of CSE induced substantially greater Th17 cells at both sites (Figure 2A). The effect was greatest in lung tissue. Interestingly, CSE had no effect on Th1 priming. Analysis of transcripts in lung tissue by quantitative real time PCR also showed greater *Il17a* transcription and no effect on *Ifng* transcripts in mice that had been given OVA with CSE (Figure 2B).

**Figure 3:**
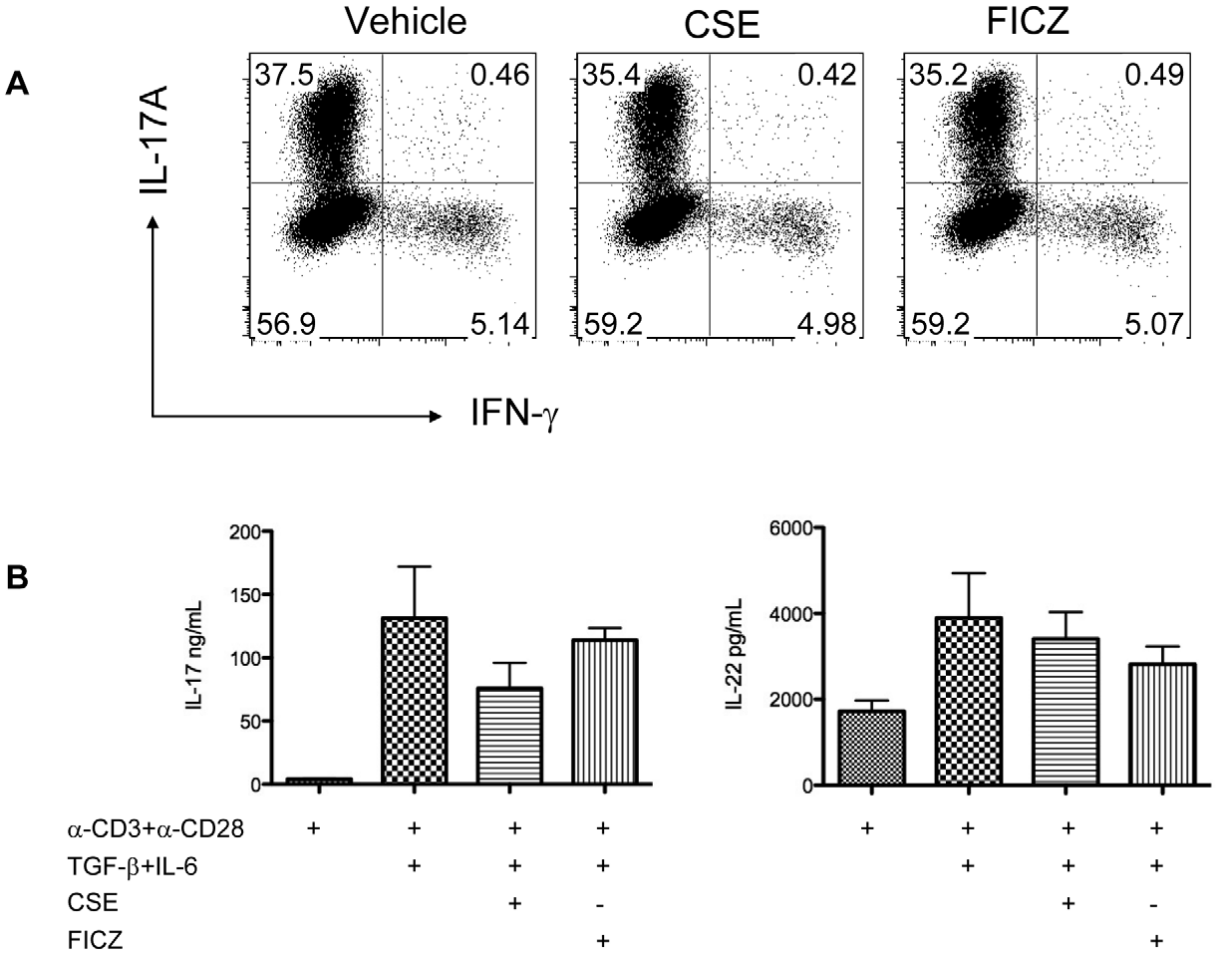
Requirement of AhR for enhanced *in vitro* Th17 polarization by cigarette smoke extract. Naïve CD4+ T cells from *Ahr−/−* spleen were activated by plated bounded anti-CD3 and anti-CD28 in the presence of TGF-b, IL-6, anti-IFN-γ and anti-IL4. Where indicated, 0.5% CSE or 200 nM FICZ were added into the culture. On day 3, cells were harvested and intracellular cytokines were analyzed by FACS (A). Tissue culture supernatants were harvested from *in vitro* polarized Th17 cells from *Ahr−/−* spleen for secreted IL-17 by Luminex and IL-22 by ELISA (B, n = 5 per group, * denotes p<0.05 compared to vehicle-HBSS).

As CSE contains aryl hydrocarbon receptor ligands, we tested the requirement for Ahr in CSE mediated Th17 *in vitro*. Naïve splenic CD4 T-cells from *Ahr^−/−^* mice could be differentiated into Th17 cells in the presence of TGF-β and IL-6; however, in contrast to WT cells (Figure 1), CD4 T-cells from *Ahr^−/−^* mice failed to show increased Th17 differentiation with CSE or FICZ (Figure 3).

### RNA-seq analysis Human Lung Epithelial cells

Polarized human bronchial epithelial (HBE) cells express IL-17RA on their basolateral surface, and the induction of G-CSF and CXCL1 by IL-17 only occurs when the ligand is applied basolaterally and not apically [22], [23]. Previously, a subset of basal cells expressing aquaporin 3 (AQP3) were found to have multi-potential differentiation characteristics in xenograft assays [23]–[25]. To determine if expression of IL-17RA, IL-17RC, and IL-22R was localized to AQP3^+^ cells, we used flow cytometry. Approximately 9–15 percent of AQP3^−^ cells stained positive for IL-17RA, IL-17RC or IL-22R (Table 1). By comparison, a significantly higher percentage of AQP3^+^ cells expressed these receptors, 26–32 percent compared to the AQP3- population, demonstrating preferential expression within the putative multi-potential population.

**Table 1:** Percent positivity of IL-17R and IL-22R expression on HBE cells gated on AQP3- versus AQP3+ cells.

	AQP3-	AQP3+
% pos. IL-17RA	15±3.2	26±3.8*
% pos. IL-17RC	11±1.9	32±4.3*
% pos. IL-22R	9±1.6	29±3.4*

*denotes p<0.05 compared to AQP3- gates. n = 3 donors, Mann-Whitney comparison.

Primary HBE cells from 3 separate donors were stained anti-AQP3, anti-IL22R, anti-IL-17RA, or anti-IL-17RC. Cells were gates on AQP3 and the percentage that stained for IL-17RA, IL-17RC, or IL22R are shown in Table 1 as mean percentages ± SEM.

**Figure 4:**
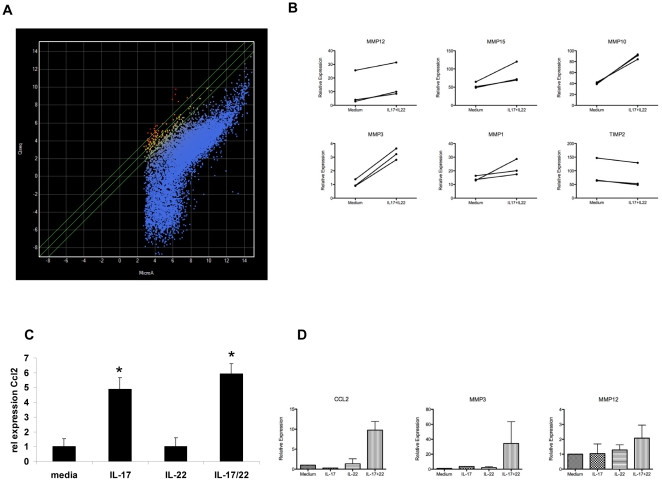
Th17 cytokines induce MMP expression in HBE cells. (A) Primary HBE cells were stimulated with media or a combination of 10/-50 ng/ml IL-22 and 50 ng/ml of IL-17A for 48 hours (n = 3 donors per condition). RNA was harvested and subjected to hybridization on Hu-133 arrays for RNA sequencing as described in the Methods. Transcript abundance or hybridization intensity was measured on normalized data in Qseq software (Lasergene). Scatter plot analysis of normalized transcript abundance for Affymetrix (x-axis) and RNA-seq (y-axis) are shown. (B) Relative gene expression of MMPs and TIMP2 from RNA-seq data. (C) Up-regulation of *Ccl2* by IL-17 and IL-22 combination treatment in primary HBE cells as measured by RNAseq. (D) CCL2, MMP3 and MMP12 gene expression were analyzed by real-time RT-PCR.

Previously, we showed that IL-22 increases the clonogenic potential of HBE cells [22], and microarray studies demonstrated an additive induction of *CSF3* and *CXCL1* among other genes following stimulation with IL-22 plus IL-17 compared to IL-17 alone [22]. Using a false discovery rate (FDR) of p<0.05, treatment with IL-22 alone induced fewer than 7 genes [22]. We postulated that the paucity of genes regulated by IL-22 could be due to a limitation of the hybridization based array approach, and thus we performed polyA+ RNA deep sequencing of three HBE donors treated with IL-17, IL-22, or both. Qseq software (Lasergene) was used to determine the relative transcript abundance. In addition, we analyzed samples that had been hybridized on Affymetrix Hu-133 arrays. Relative gene expression assayed by normalized Affymetrix signal intensity was then plotted versus the relative transcript abundance obtained by RNA sequencing (Fig. 4A; x-axis versus y-axis, respectively). When relative transcript abundance is high, the two platforms have a linear relationship with one another. However, for genes that have lower transcript abundance, the Affymetrix hybridization based methodology reaches an asymptotic relationship (with relative gene expression levels of approximately 7 or lower). In contrast, RNAseq continued to have broad dynamic range (Figure 4A), demonstrating a much higher sensitivity than microarray platforms.

We next determined if IL-17 and/or IL-22 induces genes that are involved in the pathogenesis of COPD. Specifically, we analyzed expression of matrix metalloproteinases (MMPs) that can mediate tissue degradation, and CCL2 ligands that mediate macrophage recruitment to the lung [17]. The combination of IL-17 plus IL-22 increased the expression of MMP12, MMP15, MMP10 and MMP3 in all three HBE donors samples (Figure 4B). These changes in MMP expression were not detectable using hybridization microarray technology (data not shown). We also observed a significant reduction in tissue inhibitor of metalloproteinases (TIMP) 2 expression in HBE cells treated with IL-17 and IL-22. Notably, dual cytokine treatment resulted in a significant induction of CCL2 (Figure 4C), but not CCL3 or CCL5 (data not shown). The up-regulation of *Ccl2*, *Mmp3* and *Mmp12* were confirmed by real-time RT-PCR (Figure 4D), demonstrating a correlation between Th17 cytokines and the upregulation of genes involved in COPD.

### IL-17RA regulates expression of CCL2, *Mmp9*, *Mmp12*, and the development of emphysema *in vivo*

**Figure 5:**
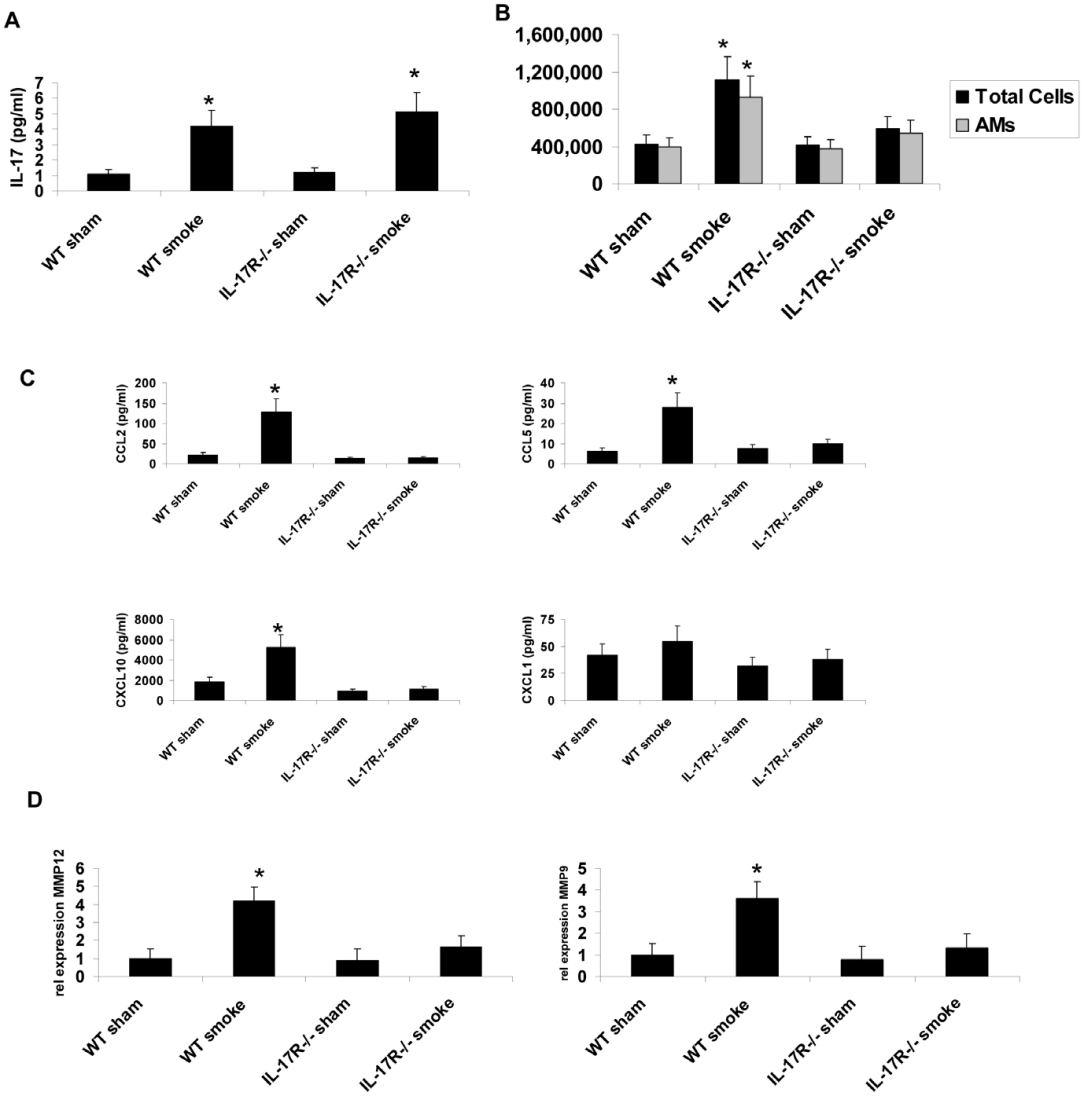
Requirement of IL-17RA for smoke induced lung inflammation. (A) BAL levels of IL-17 after six months of cigarette smoke exposure (n = 5–6 per group, * denotes p<0.05 compared to WT sham). (B) BAL total cells and alveolar macrophage numbers after 6 months of smoke exposure. (n = 5–6 per group, * denotes p<0.05 compared to WT sham) (C) Levels of CCL2, CCL5, CXCL10 and CXCL1 in lung homogenate after six months of cigarette smoke exposure (n-5–6 per group, * denotes p<0.05 compared to WT sham). (D) Expression of transcripts for MMP12 and MMP9 using RT^2^ Profiler PCR Array. The delta C_t_ values were calculated by individual gene C_t_ value minus that of the associated housekeeping gene and expressed as relative gene expression compared to the WT sham group (n-5–6 per group, * denotes p<0.05 compared to WT sham).

**Figure 6:**
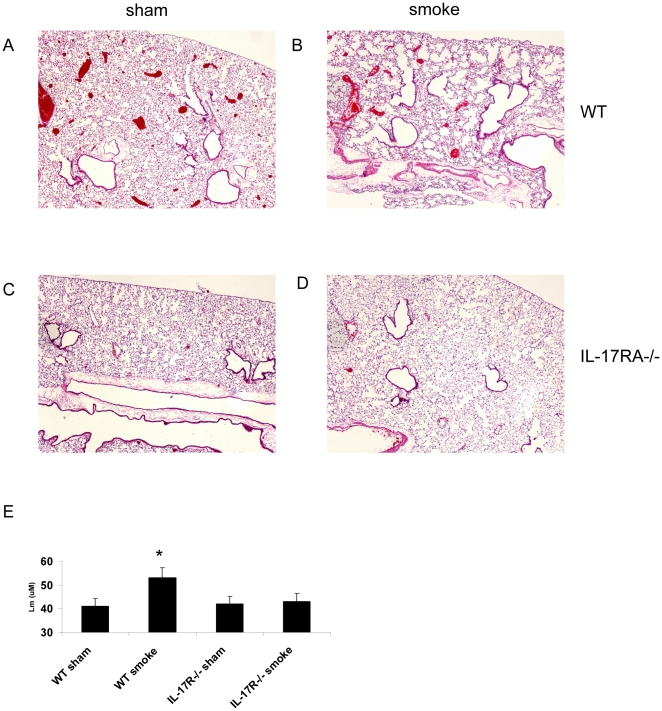
Requirement of IL-17RA for emphysema. Representative histological sections of mouse lungs stained with hematoxylin and eosin after six months of exposure to air (sham exposed) (A,B) or cigarette smoke (C,D) demonstrate C57BL/6 mice develop alveolar enlargement (C) as opposed to IL-17RA−/− mice (D). (E) Enlargement of airspaces was quantified using the mean linear intercept method. This method determines the parenchymal surface area relative to lung volume. An increase in Lm represents an increase in the alveolar space as seen in the WT smoking mice versus sham air. This increase in Lm was not seen in the IL-17A−/− mice over sham air controls. All lung tissues were inflated to 25cm water pressure with neutral buffered formalin. Original magnification: x 50 of representative sections.

To determine if IL-17RA was required for expression of the genes identified by our RNAseq *in vivo* after cigarette smoke exposure, we analyzed expression of these genes by quantitative real time PCR in WT or IL-17RA^−/−^ mice exposed cigarette smoke for 6 months as previously described [17]. IL-17 protein levels were significantly elevated in the BAL of mice exposed to smoke compared to sham controls (Figure 5A). We also noted similar induction of IL-17 protein in IL-17RA^−/−^ mice exposed to smoke (Figure 5A). Moreover, smoke-exposed IL-17RA KO mice had significantly lower total cells as well as macrophage numbers in BAL compared to WT mice (Figure 5B). Reductions in alveolar macrophage numbers in BAL were associated with a significantly reduced level of the chemokines CCL2, CCL5, and CXCL10, but not CXCL1 (Figure 5C). Analysis of MMP expression by quantitative real time PCR also showed reduced levels of MMP12 and MMP9 in smoke exposed IL-17RA^−/−^ mice compared to WT mice (Figure 5D). We next examined tissue emphysema in lungs inflated to 20 cm H_2_O. Sham exposed mice showed normal lung architecture (Figure 6A and B) as well as values for mean alveolar distance that were in the normal range (Figure 6E). WT smoke exposed mice showed significant airspace enlargement compared to the sham control (Figure 6C). In contrast IL-17RA^−/−^ mice showed significantly less tissue emphysema (Figure 6D), which was also associated with a reduced mean linear intercept (Figure 6E) that approached the level of sham exposed mice.

## Discussion

The development of tissue emphysema due to cigarette smoke exposure in mice requires macrophages and the expression of matrix metalloproteinase 12 and 9 [17], [26], [27]. Interestingly, the intratracheal administration of CCL2 can augment emphysema but not in MMP12^−/−^ mice [17], suggesting that MMP12 rather than macrophages per se is critical for emphysema [17]. IL-17 has been shown to be an important inducer of CCL2, and mice overexpressing IL-17A or IL-17F show elevated CCL2 in the lung [26], [27]. There has been much interest in the role of the adaptive immune response in COPD since it has been recently reported that COPD in humans is associated with the development of lymphoid follicles in the lung [3]. Moreover, patients with COPD have evidence of anti-elastin antibodies and Th1 responses, suggesting an autoimmune component [28]. Furthermore, mice deficient in CD8^+^ T-cells are resistant to cigarette smoke induced emphysema whereas CD4^+^ T-cell deficient mice are not [29].

Recently we have shown that IL-17RA is critical for germinal center formation in BXD2 mice as well as lymphoid follicles in mice with EAE [30], [31]. These data suggest that IL-17RA may play a role in lymphoid follicle development as well as other pathological features in COPD. In further support of this hypothesis, IL-17 producing cells are increased in the lungs of patients with COPD [4], [32]. Cigarette smoke is a complex mixture of particulates that contain TLR agonists as well as aryl hydrocarbon ligands [18], [19]. Addition of CSE to T-cells undergoing Th17 differentiation augmented the percentage of IL-17 and IL-22 producing cells as well as the transcription factor *Rorc*. Importantly, the addition of CSE to OVA significantly increased the number of Th17 cells *in vivo* as well. We did not observe a significant effect on Th1 responses. One mechanism by which cigarette smoke acts as a Th17 adjuvant is via Ahr, as T-cells form Ahr^−/−^ mice did not show increased Th17 differentiation with CSE or the known Ahr ligand FICZ. Given the fact that IL-17 over expression increases CCL2 expression, we hypothesized that IL-17RA^−/−^ mice would have reduced macrophage recruitment and reduced cigarette smoke induced emphysema. After 6 months of cigarette smoke exposure we found that IL-17RA was critical for the induction of CCL2, macrophage recruitment, MMP12 expression, and emphysema in mice. We also observed a significant reduction of the CCR3 ligand CCL5 in IL-17RA KO mice and CXCR3 ligands like CXCL10 have also been shown to induce MMP12 expression in macrophages [29], [33]. Taken together, these data suggest that IL-17RA expression may be affecting two independent processes that are required for the development of emphysema, namely macrophage recruitment via CCL2 and induction of MMP12 by CXCL10.

It is important to note that IL-17RA has at least three ligands including IL-17A, IL-17F, and IL-25. Both IL-17A and IL-17F can regulate CCL2 and CXCL10 expression [26], [27] and thus the individual contributions of these two IL-17 family members needs to be determined. We did not observe increases in IL-25 in this model (data not shown) or an increase in eosinophils which make IL-25, and therefore IL-17RB signaling is unlikely to play a significant role in this phenotype. The specific roles of IL-17A and IL-17F are being investigated in ongoing studies using specific knockout mice.

Although the expression of Ahr was required for the effect of CSE on *in vitro* Th17 differentiation, other factors such as LPS, lipopeptides or other activators of innate immunity likely play critical roles in priming Th17 responses *in vivo*. For example, smoke could also activate the inflammasome and processing of IL-1β, another critical factor in regulating Th17 differentiation [34], [35]. We hypothesize that major target cells of IL-17RA dependent signaling are epithelial cells, as prior studies using in situ hybridization have shown they are crucial for IL-17-induced chemokine expression [22]. IL-17 regulates CCL2 in both human and mouse lung epithelium, and we hypothesize that CCL2 is required for the increased number of macrophages in smoke exposed WT mice as opposed to IL-17RA^−/−^ mice. Mechanistically, it has been shown that IL-17 stabilizes the half-life of chemokine mRNAs [36], and this may explain the increase in transcripts for CCL2. In summary, cigarette smoke is a selective Th17 adjuvant in part via the Ahr receptor. This may also in part explain the known association of tobacco smoking and autoimmune diseases such as systemic lupus erythematosus [14], rheumatoid arthritis [15] and inflammatory bowel diseases [16]. *In vivo* cigarette smoke induces CCL2 in the lung in an IL-17RA dependent manner, and IL-17RA is essential for smoke induced macrophage recruitment and emphysema. These data demonstrate that antagonism of the Th17 pathway may be a therapeutic target in COPD.

## Methods

### Ethics Statement

All procedures were performed according to the University of Pittsburgh and LSU Health Sciences Center IACUC guidelines (protocols 168 and 2660 respectively). Studies with primary human bronchial epithelial cells were approved by the University of Pittsburgh IRB, protocol number IRB970946.

### Mice and intratrachel administrations

C57BL/6 (B6), B6.Cg-Tg(TcraTcrb)425Cbn/J (OTII) mice were purchased from The Jackson Laboratory. 50 µg Ovalbumin (Sigma) was given to isoflurane anesthetized mice in sterile PBS (50 µl) by oropharyngeal aspiration-tongue pull technique together with 50 µL HBSS or 100% cigarette smoke extract. *Il17Ra^−/−^* mice on the B6 background were provided by Amgen (Seattle, WA). AhR KO mice were obtained from Dr Frank Gonzalez (National Cancer Institute, Bethesda, MD).

### Preparation of CSE

Research-grade cigarettes (1R3F) with a filter from the Kentucky Tobacco Research and Development Center at the University of Kentucky (Lexington, KY) were smoked to 0.5 cm above the filter in a fume hood, using a modification of the method developed by Carp and Janoff [37]. CSE was prepared by bubbling smoke from 8 cigarettes into 10 ml of HBSS at a rate of 1 cigarette/min. The pH of the HBSS was adjusted to 7.4 and the medium was sterile filtered with a 0.45 um filter (Corning). The 10 ml HBSS was then used as 100% CSE.

### *In vitro* differentiation of Th17 cells

CD4+ T cells from the spleens and lymph nodes of B6 mice were enriched by negative selection using magnetic beads (Miltenyi Biotec). CD4+CD62L+CD25– cells were sorted on a FACSAria (BD Biosciences); purity was consistently >99%. Cells were activated by plate-bonded anti-CD3 (3 µg/mL) and anti-CD28 (3 µg/mL) in RPMI medium supplemented with 5 ng/ml porcineTGF-β, 40 ng/ml rmIL-6, 5 µg/ml anti-IL-4, and 5 µg/ML anti-IFN-γ. Where indicated 0.5% CSE or 200 nM FICZ (BioMol) were added to the culture. On Day3, tissue culture supernatants were harvested for secretary cytokine analysis and cells were harvested for intracellular cytokine analysis.

### Isolation of murine lung single cells for ICS

Lungs are isolated and minced with forceps and small scissors and digested with 1-2 mg/mL Collagenase (Sigma) for 90 min at 37°C. Digested tissues were passed through a sterile 70 µm filter (BD Falcon) to get a single cell suspension. After washing, the cells are ready for FACS or *in vitro* stimulation.

### Flow cytometric analysis

*In vitro* polarized T cells or single cells from mouse lung were stimulated for 5–6 h with 50 ng/ml PMA (Sigma) and 750 ng/ml ionomycin (Sigma). After 1 h, 1 µL/mL GolgiStop (BD Pharmingen, San Diego, CA) was added to block cytokine secretion. Cells were surface stained for 15–30 min at 4°C with anti-CD4 mAb (RM45; BD Pharmingen, San Diego, CA) in PBS supplemented with 1% BSA and 0.2% sodium azide. Cells were then fixed and permeabilized with Cytofix/Cytoperm (BD Pharmingen, San Diego, CA) and stained intracellularly with anti-IL-17, anti-IL-22 and anti-IFN-γ (XMG1.2) (eBioscience, San Diego, CA). Samples were acquired on a FACSAria or LSR-II flow cytometer and data analysis was conducted using FlowJo software (Treestar). HBE cells were stained with anti-AQP3 followed by anti-IL-22R anti-IL-17RA or anti-IL-17RC (mouse anti human IL-17RC generously provided by S. Levin, Zymogenetics).

### RNA-seq analysis

HBE cells, purchased from Lonza (Walkersville, MD), were cultured according to Gray and colleagues [38]. Primary human bronchial epithelial cells were treated with IL-17 (50 ng/mL) and/or IL-22 (10–50 ng/mL) for 48 h. Total RNA was isolated using TRIZOL Reagent (Invitrogen; Carlsbad, CA) and RNA integrity was determined with the Agilent 2100 Bioanalyzer (Carlsbad, CA). Samples with RINs ranging from 7.6 to 9.9 were processed with an mRNA-Seq. Sample Prep. Kit (Illumina, Inc.; San Diego, CA). Briefly, mRNA was purified from 2–5 ug of total RNA using Sera-Mag Oligo(dT) Beads, fragmented with magnesium-catalyzed hydrolysis and reverse transcribed into cDNA using random primers (Superscript II; Invitrogen). Then, cDNA underwent end repair with T4 DNA polymerase and Klenow DNA polymerase, followed by the addition of ‘A’ bases to the 3′ end, and ligation to adaptor oligos. Products from the ligation were run on a 2 percent agarose gel. A gel slice consisting of the 200 bp region (+/−25 bp) was excised and used as a template for PCR amplification. The final PCR product was purified, denatured with 2 N NaOH, and diluted to 10–12 pM prior to cluster amplification on a single-read flow cell v4, as outlined in the Single Read Cluster Generation Kit v4 (Illumina). The flow cell was sequenced on an Illumina Genome Analyzer II at the LSUHSC Genomics Core facility.

### Cigarette smoke exposure

Eight to ten weeks *Il17Ra^−/−^* female mice and their age, gender matched non-smoke controls were subjected to two unfiltered cigarettes per day (University of Kentucky), 5 days per week for 6 months, with the use of a smoking apparatus as previously described [17]. Mice tolerated smoke exposure without toxicity; carboxyhemoglobin levels after two cigarettes were 10%. Sham and non-smoke-exposed age-matched littermates were used as controls. Here and previously, sham chamber-exposed mice were equivalent to non-smoke-exposed mice.

### Bronchoalveolar lavage

Mice were anesthetized with Ketamine. A tracheal cannula was inserted into the upper cervical trachea through a tracheotomy. The lungs were lavaged with 0.5-ml aliquots of warmed PBS + 0.5 mM EDTA up to 11 ml. The first ml of recovered bronchoalveolar lavage (BAL) fluid was centrifuged at 500 *g* and the supernatant was stored at −80°C for subsequent cytokine ELISAs. The remaining cell pellet was combined with the other lavage fluid and analyzed for total cell count followed by differential cell counts performed in cytospins and stained with Wright-Giemsa (Baxter McGaw Park).

### Measurement of cytokines

Right lung tissue was harvested after BAL and was homogenized for cytokine assay. All cytokines were measured by multiplex analysis using Luminex (Millipore) on the Bioplex reader (Bio-Rad).

### Morphometry

Following smoke exposure, each mouse was sacrificed and the lung was inflated by instilling 3% gluteraldehyde in 0.1 mol/L cacodylate buffer to 25 cm H_2_0 (for 15 minutes), and then ligated and removed. Inflated lungs were fixed for 48 hours before embedding in paraffin. Serial sagittal sections were obtained. Mid-sagittal sections stained with hematoxylin and eosin (H&E) were used to calculate the mean linear intercepts as previously described [[17]].

### MMP gene expression assay

Mouse extracellular matrix metallopeptidase gene expression was measured by using RT^2^ Profiler PCR Array kit following manufacturer's protocol (SABiosiences). Briefly, total lung tissue was harvested and total RNA was extracted using TRIzol (Invitrogen). First strand cDNA was synthesized after genomic DNA was eliminated. cDNA was then used for real-time PCR for MMPs gene expression using RT^2^ Profile PCR Array primer plate and SYBR green qPCR master mix. The delta C_t_ values were calculated by individual gene C_t_ value minus that of the associated housekeeping gene.

### Statistical Analysis

Comparison between means was performed with analysis of variance (ANOVA) followed by Fisher's follow-up testing or by Mann-Whitney non parametric analysis. *p*<0.05 was considered to represent a significant difference.
